# Exosome-mediated uptake of mast cell tryptase into the nucleus of melanoma cells: a novel axis for regulating tumor cell proliferation and gene expression

**DOI:** 10.1038/s41419-019-1879-4

**Published:** 2019-09-10

**Authors:** Fabio Rabelo Melo, Sebastin Santosh Martin, Christian P. Sommerhoff, Gunnar Pejler

**Affiliations:** 1 Uppsala University, Department of Medical Biochemistry and Microbiology, Uppsala, Sweden; 2 Institute of Laboratory Medicine, University Hospital, LMU Munich, Munich, Germany; 3 Swedish University of Agricultural Sciences, Department of Anatomy, Physiology and Biochemistry, Uppsala, Sweden

**Keywords:** Cell growth, Melanoma, Preclinical research

## Abstract

It is well established that mast cell accumulation accompanies most malignancies. However, the knowledge of how mast cells functionally impact on tumors is still rudimentary. Here we addressed this issue and show that mast cells have anti-proliferative activity on melanoma cells and that this effect is dependent on tryptase, a tetrameric protease stored in mast cell granules. Mechanistically, tryptase was found to be endocytosed by melanoma cells as cargo of DNA-coated exosomes released from melanoma cells, followed by transport to the nucleus. In the nucleus, tryptase executed clipping of histone 3 and degradation of Lamin B1, accompanied by extensive nuclear remodeling. Moreover, tryptase degraded hnRNP A2/B1, a protein involved in mRNA stabilization and interaction with non-coding RNAs. This was followed by downregulated expression of the oncogene EGR1 and of multiple non-coding RNAs, including oncogenic species. Altogether, these findings establish a new principle for regulation of tumor cell proliferation.

## Introduction

Mast cells (MCs) are hematopoietic cells of the immune system, classically well known for their detrimental impact on allergic reactions^1–3^. In addition, there are numerous reports showing that MCs frequently populate tumors, often in large numbers (reviewed in refs. ^4–8^). In many clinical studies a positive correlation between MC presence and poor disease outcome has been reported, and this has been taken as an indication that MCs can worsen the disease^4–8^. There are also several studies in various animal models implicating MCs as detrimental players in malignant conditions^9–11^. On the other hand, there are also frequent examples of malignancies where MC presence correlates with good prognosis, i.e., that MCs might be protective^4–8^. However, although these studies collectively implicate MCs in various aspects of malignant settings, there is still very little knowledge regarding the molecular mechanism(s) by which MCs can affect tumor growth.

MCs are characterized by a high content of lysosome-like secretory granules containing large amounts of various preformed bioactive compounds, including histamine, serotonin, various cytokines and growth factors, serglycin proteoglycans, and large amounts of MC-restricted proteases^12^. The latter include tryptases, chymases, and carboxypeptidase A3 (CPA3), of which tryptase and chymase are serine proteases whereas CPA3 is Zn-containing metalloprotease^13,14^. Previous studies have suggested that the MC-restricted proteases can contribute to the manifestations of numerous pathologies^12,15^, but their possible contribution in malignant settings has received little attention. In a previous study we approached this issue and showed that the combined absence of tryptase, chymase, and CPA3 can lead to increased tumor progression in a melanoma model^16^. There is also clinical evidence suggesting a correlation between the presence of MC proteases and good outcome in melanoma^17,18^. However, there is no mechanistic insight into how MC proteases could exert such anti-tumor effects. Here we addressed this issue and show that one of the MC proteases, tryptase, suppresses the proliferation of melanoma cells. The underlying mechanism involved binding of tryptase to DNA-coated exosomes released from the melanoma cells, followed by uptake into the melanoma cells and transport into the nucleus. In the nucleus, tryptase degraded structural and RNA-regulating proteins, accompanied by remodeling of the nuclear structure and effects on the expression of non-coding RNAs. Taken together, these findings identify a novel principle for regulation of tumor cell proliferation.

## Results

### Suppression of mouse melanoma cell expansion by MCs is dependent on tryptase

**Fig. 1:**
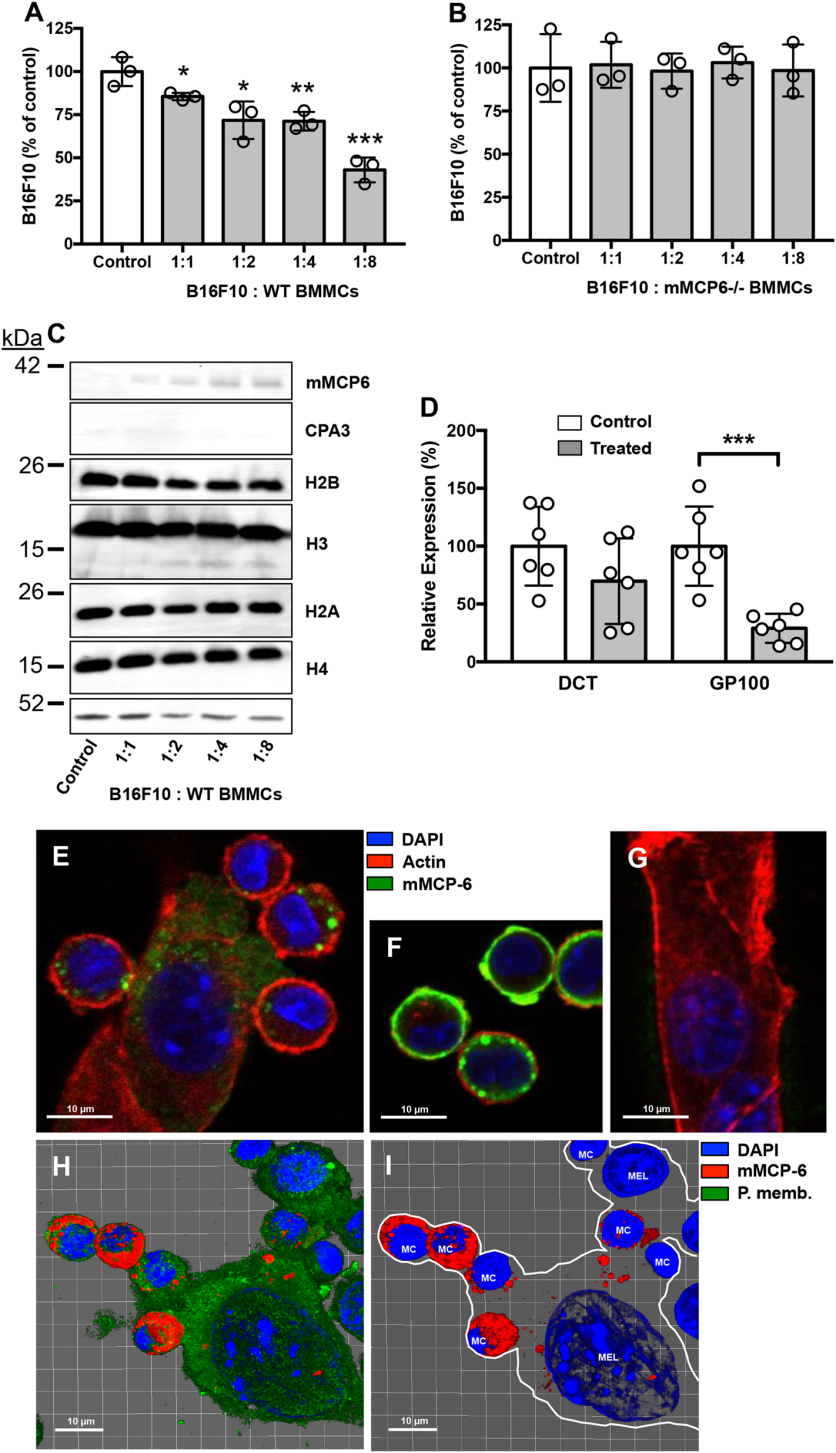
Mast cell tryptase suppresses melanoma cell expansion and deliver tryptase into the nucleus of melanoma cells. WT (**a**) or tryptase-deficient (mMCP6^−/−^) (**b**) mouse mast cells were co-cultured with mouse melanoma cells (B16F10) at the ratios indicated, followed by quantification of viable cells using Trypan Blue exclusion. **c** After co-culture, melanoma cells were separated from mast cells and were analyzed by Western blotting for cell-associated tryptase (mMCP6), CPA3, and core histones H2B, H3, H2A, and H4. Actin was used as loading control. **d** After co-culture of mast cells and melanoma cells, cells were harvested and analyzed by qPCR for expression of melanoma markers DCT and GP100. The displayed results are from individual experiments, representative of at least three experiments. Data in (**a**) and (**d**) are given as mean values ± SEM; *p ≤ 0.05, **p ≤ 0.01, ***p ≤ 0.001. **e** Confocal microscopy image showing physical contact between mast cells (MC) and melanoma cells (Mel) after co-culture. **f**, **g** Controls showing mast cells (**f)** and melanoma cells (**g**) alone. **h**, **i** Confocal images were analyzed with IMARIS software to generate 3-D-images of the interaction between mast cells and melanoma cells. Note the presence of mast cell tryptase in the nucleus of melanoma cells after mast cell:melanoma cell co-culture

In order to evaluate the impact of MCs on melanoma we first performed experiments where mouse bone marrow-derived MCs were co-cultured with mouse melanoma cells (B16F10), followed by assessment of cell numbers. As seen in Fig. 1a, incubation of the melanoma cells with wild-type MCs caused a substantial and significant reduction in the numbers of melanoma cells, suggesting that MCs have a suppressing impact on the melanoma population. However, when co-culturing the melanoma cells with MCs lacking tryptase (mMCP6^−/−^), no reduction of melanoma cell numbers was seen (Fig. 1b), indicating that MC tryptase is the key factor regulating melanoma growth. Importantly, the absence of tryptase does not affect the ability of MCs to store chymase or CPA3, nor does the absence of tryptase affect the ability of MCs to undergo immunological activation^19–21^. Hence, these findings most likely represent direct effects of tryptase, rather than confounding effects related to the knockout of mMCP6.

### Tryptase is delivered from MCs to the nucleus of mouse melanoma cells

To decipher how MC tryptase affects the melanoma cells we first assessed whether tryptase released from MCs interacts physically with the melanoma cells. As seen in Fig. 1c (left lane), tryptase was as expected not detected in melanoma cells that had not been co-cultured with MCs. However, after co-culturing melanoma cells with MCs, a band corresponding to tryptase (mMCP6) was detected in the melanoma cell population, suggesting that tryptase indeed is associated with the melanoma cells. In contrast, CPA3 was not detected in the melanoma fraction (Fig. 1c), suggesting that there is specific binding of tryptase to melanoma cells rather than a general transfer of granule contents from MCs to melanoma cells. In previous studies we showed that MC tryptase, in addition to its location within the MC secretory granules, also can be found within the nucleus and has the ability to truncate nucleosomal core histones^22,23^. This prompted us to investigate whether tryptase taken up by melanoma cells could execute core histone processing. Indeed, as depicted in Fig. 1c, truncation of histone 3 (H3) in the melanoma cells was seen after co-culture with MCs, whereas the other core histones were not affected (Fig. 1c). This suggests that tryptase, secreted from the MCs, reaches the nucleus of the melanoma cells in an enzymatically active form and there executes core histone processing.

We next evaluated the impact of MCs on the expression of melanoma-specific genes: DCT and GP100. These analyses revealed that co-culture of the melanoma cells with MCs caused a profound downregulation of the expression of GP100, indicating that MCs affect the phenotype of the melanoma cells (Fig. 1d). Next, we used microscopy to investigate the interaction between MCs and melanoma cells. As seen in Supplementary Video 1, MCs were attracted to the tumor cells, often with numerous MCs moving towards individual melanoma cells. Interaction between the MCs and melanoma cells was also visualized by confocal microscopy analysis, showing close physical contact between the respective cell types (Fig. 1e). Notably, MCs and melanoma cells could be readily distinguished by morphological criteria, and also by the lack of tryptase expression in melanoma cells (Fig. 1f, g). From the confocal analysis it was evident that MCs form close contacts with the melanoma cells and that tryptase is delivered from the MCs to the interior of the melanoma cells (Fig. 1e). To obtain further insight into the interaction of MCs with melanoma cells we performed a 3-dimensional analysis of the MC/melanoma co-cultures. This analysis confirmed the close physical association of MCs with the melanoma cells, and also revealed that tryptase was found in the melanoma cells that had been co-cultured with MCs (Fig. 1h, i and Supplementary Video 2).

### Human tryptase causes morphological effects and reduction in cell numbers of human melanoma cells

**Fig. 2:**
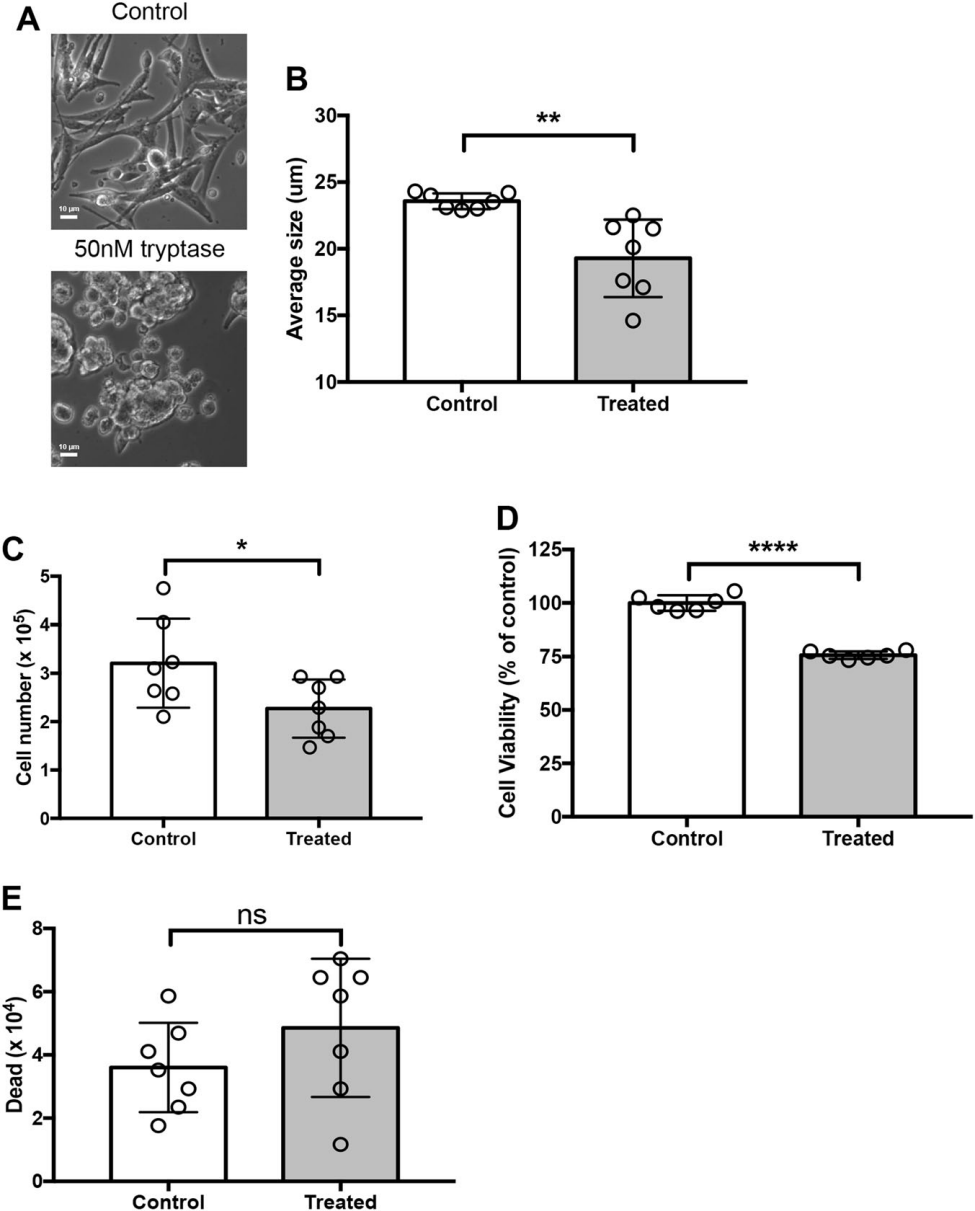
Human recombinant tryptase affects human melanoma cell phenotype and expansion. Human melanoma cells (MEL526) were incubated for 48 hours with 50 nM human recombinant tryptase. **a** Microscopy analysis revealing gross morphological alterations in melanoma cell treated with tryptase. **b** Assessment of cellular size showing that tryptase causes a reduction in melanoma cell size. **c**, **d** Quantification of viable cells using Trypan Blue exclusion (**c**) and CellTiter-Blue® viability probe (**d**). **e** Dead cells were quantified using Trypan Blue positivity. Data in (**b**–**e**) are given as mean values ± SEM; *p ≤ 0.05, **p ≤ 0.01, ****p ≤ 0.0001

To obtain more mechanistic insight into how MC tryptase affects melanoma cells we next performed experiments in which recombinant human tryptase was incubated with human melanoma cells (MEL526). These experiments revealed that human melanoma cells underwent striking morphological changes after incubation with tryptase, with a pronounced shift from an extended morphology to a more rounded appearance (Fig. 2a), this being associated with a significant decrease in cellular size (Fig. 2b). The morphological effects were seen after long-term (24 h) incubation of tryptase over a range of concentrations (25–75 nM; shown for 50 nM). It was also observed that incubation of melanoma cells with tryptase caused a reduction in the number of melanoma cells, as evidenced by quantification of the cells by both Trypan Blue exclusion (Fig. 2c) and by enumerating cells using a viability probe (Fig. 2d).

### Tryptase causes reduced proliferation in human melanoma cells

**Fig. 3:**
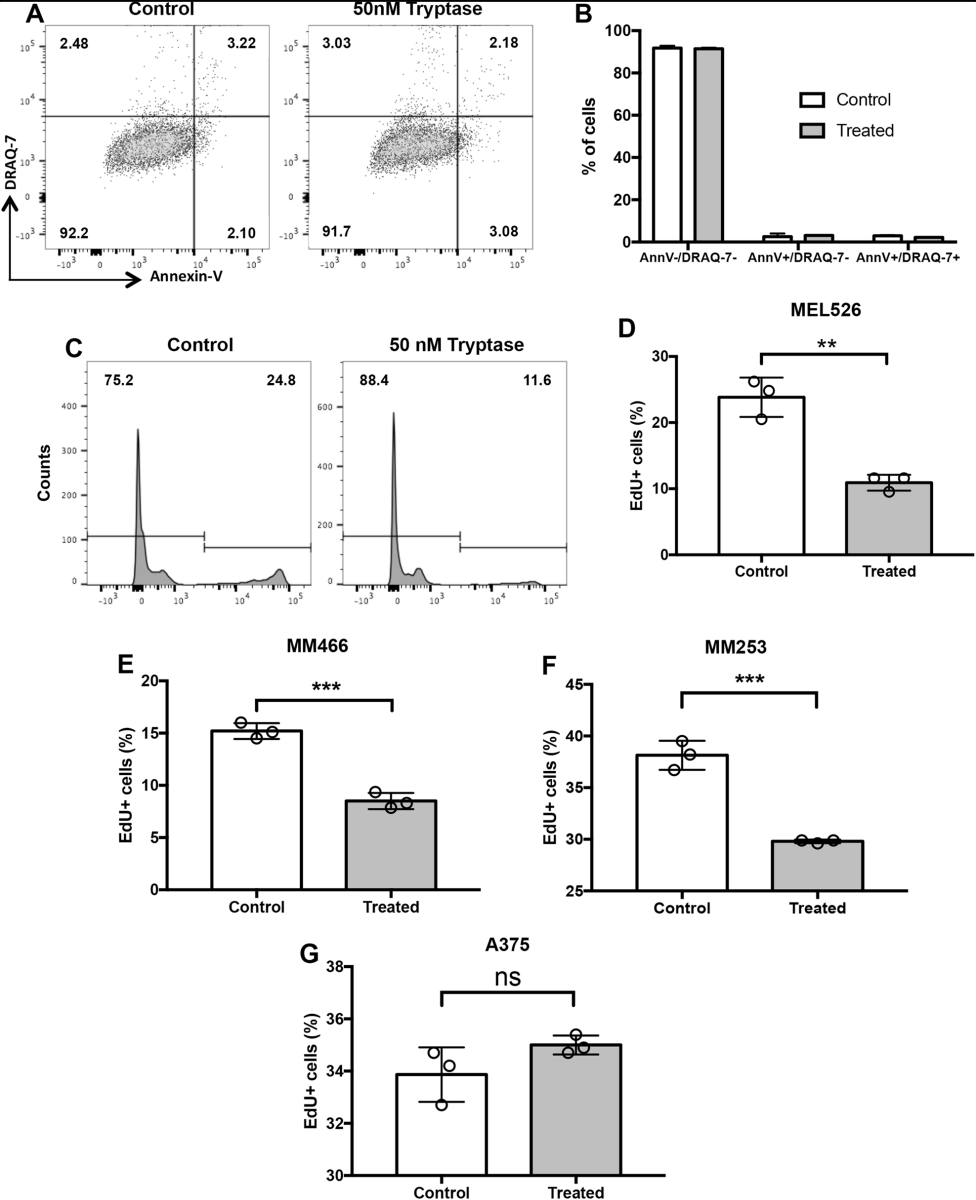
Tryptase causes decreased proliferation of melanoma cells. Melanoma cells (MEL526) were incubated for 48 hours with 50 nM human tryptase. **a** After incubation, melanoma cells were stained with Annexin V/Draq7 to monitor apoptosis (Annexin V+/Draq7−) and necrosis (Annexin V+/Draq7+). Note that tryptase does not induce apoptosis/necrosis in melanoma cells. **b** Quantification of the Annexin V/Draq7 staining. **c** Tryptase-treated melanoma cells (MEL526) were assessed for proliferation through EdU staining. Note the decreased EdU staining after treatment of melanoma cells with tryptase. **d**–**g** Quantification of EdU staining of MEL526 (**d**), MM466 (**e**), MM253 (**f**), and A375 (**g**) melanoma cells. Data in (**b**, **d**–**g**) are given as mean values ± SEM; **p ≤ 0.01, ***p ≤ 0.001; ns, not significant

One possible explanation for the reduction in cell numbers could be that tryptase has cytotoxic effects on the melanoma cells. However, incubation of melanoma cells with tryptase did not result in a statistically significant increase in numbers of dead cells, as assessed by Trypan Blue positivity, although a trend of increased Trypan Blue positivity was noted (Fig. 2e). This was also supported by Annexin V/Draq7 staining, revealing that tryptase did not cause any significant elevation in the proportion of apoptotic or necrotic cells (Fig. 3a, b). An alternative explanation could be that tryptase suppresses the proliferation of the melanoma cells. To address this possibility, non- and tryptase-treated melanoma cells were stained with EdU, a thymidine analogue, followed by flow cytometry analysis. As seen in Fig. 3c, d, a robust peak representing EdU-positive (proliferating) cells was seen in non-treated cells. However, after incubation with tryptase, the corresponding peak was markedly diminished (Fig. 3c, d). Hence, these data indicate that tryptase suppresses the proliferation of melanoma cells, without inducing apoptosis. For comparison, the melanoma cells were also incubated with recombinant human chymase. However, chymase at concentration range of 25–75 nM did not affect the proliferation or morphological features of the melanoma cells (Supplementary Fig. 1). To assess the effects of tryptase on melanoma cells beyond its effect on the MEL526 line, we also assessed the effect of tryptase on other melanoma cell lines. For this purpose we evaluated the MM466, MM253 and A375 cell lines. As for MEL526 cells, tryptase suppressed the proliferation of the MM466 and MM253 lines (Fig. 3e, f). In contrast, tryptase had no effect on the proliferation of the A375 line (Fig. 3g).

### Tryptase is taken up to the nucleus of melanoma cells and causes nuclear remodeling

**Fig. 4:**
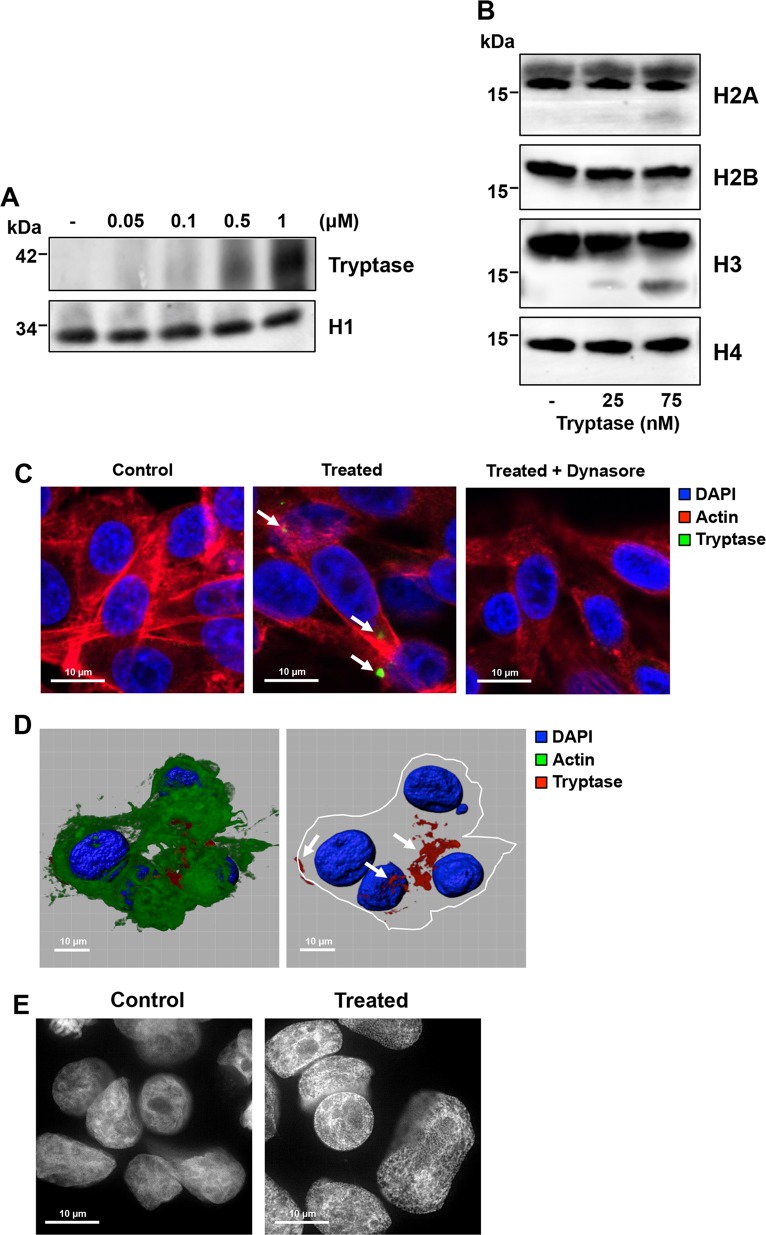
Tryptase is taken up into the nucleus of human melanoma cells, and affects nuclear morphology and histone processing. **a** Human melanoma cells (MEL526) were incubated with human tryptase at the indicated concentrations, followed by washing of the cells and Western blot analysis for tryptase. Histone 1 (H1) was used as loading control. **b** Western blot analysis for core histones H2A, H2B, H3, and H4 after incubation of human melanoma cells with tryptase at the indicated concentrations for 48 h. Note the clipping of H3 after treatment of melanoma cells with tryptase. **c** Confocal analysis showing uptake of tryptase into melanoma cells and blocked uptake in the presence of an endocytosis inhibitor (Dynasore). **d** 3-D imaging using the IMARIS software showing the presence of tryptase in both the cytoplasm and nucleus of human melanoma cells (see also Supplementary Fig. 2). **e** DAPI staining of control and tryptase-treated human melanoma cells using super-resolution microscopy analysis. Note the extensive morphological alterations of the nucleus after tryptase treatment. Note also the presence of pores in the nuclear envelope after treatment with tryptase

To provide further insight into the mechanism by which tryptase affects the human melanoma cells we first assessed whether tryptase binds to the melanoma cells. Indeed, Western blot analysis showed that tryptase could be detected in the melanoma cell fraction, indicating that tryptase becomes associated with the cells (Fig. 4a). Next, we assessed whether tryptase can execute core histone processing in the human melanoma cells. As depicted in Fig. 4b, incubation of human melanoma cells with human tryptase resulted in truncation of H3, whereas H2A, H2B, and H4 were minimally affected. The latter findings suggest that tryptase is found within nucleus of the melanoma cells in an enzymatically active form. Indeed, confocal microscopy analysis revealed that tryptase was taken up by the melanoma cells and could be detected both in the cytoplasm and in the nucleus (Fig. 4c, d; Supplementary Fig. 2 showing a Z-stack analysis). The uptake of tryptase into the melanoma cells was completely inhibited in the presence of Dynasore, a dynamin inhibitor, indicating that tryptase is taken up through endocytosis (Fig. 4c). To further assess the impact of tryptase on the nucleus of the melanoma cells, we conducted super-resolution microscopy analysis. In the absence of exogenous tryptase, the nucleus of the melanoma cells had a smooth appearance with an intact nuclear envelope. In contrast, after incubation of melanoma cells with tryptase, the nuclear membrane displayed signs of perforation and the nuclear morphology was dramatically affected, showing a much more irregular structure (Fig. 4e). Hence, these findings indicate that human tryptase is taken up by human melanoma cells, is transported into the nucleus and there truncates core histones and induces nuclear remodeling.

### The effect of tryptase on melanoma cells requires enzymatic activity but is independent of protease-activated receptor 2

Previous research has indicated that biological effects of tryptase can be due to its ability to cleave protease-activated receptor 2 (PAR-2)^24–26^. To evaluate if the effects of tryptase on melanoma cells could be mediated by PAR-2, we exposed melanoma cells to a PAR-2 agonist. However, melanoma cells were unresponsive to the PAR-2 agonist (Supplementary Fig. 3), arguing against a major role of PAR-2 in the response of melanoma cells towards tryptase. Another scenario would be that tryptase, being a heparin-binding protein^27^, could interact with heparin-like glycosaminoglycans on the melanoma cell surface. However, the addition of excess heparin to the extracellular medium did not affect the ability of tryptase to suppress melanoma cell proliferation, arguing against this possibility (Supplementary Fig. 3). We also assessed whether the effects of tryptase were dependent on its enzymatic activity by using a selective tryptase inhibitor (Nafamostat)^28^. As seen in Supplementary Fig. 3, Nafamostat completely abrogated the effect of tryptase on melanoma cell proliferation, supporting that the effects of tryptase are dependent on its enzymatic activity.

### Tryptase is taken up into melanoma cells as cargo of melanoma-derived exosomes

**Fig. 5:**
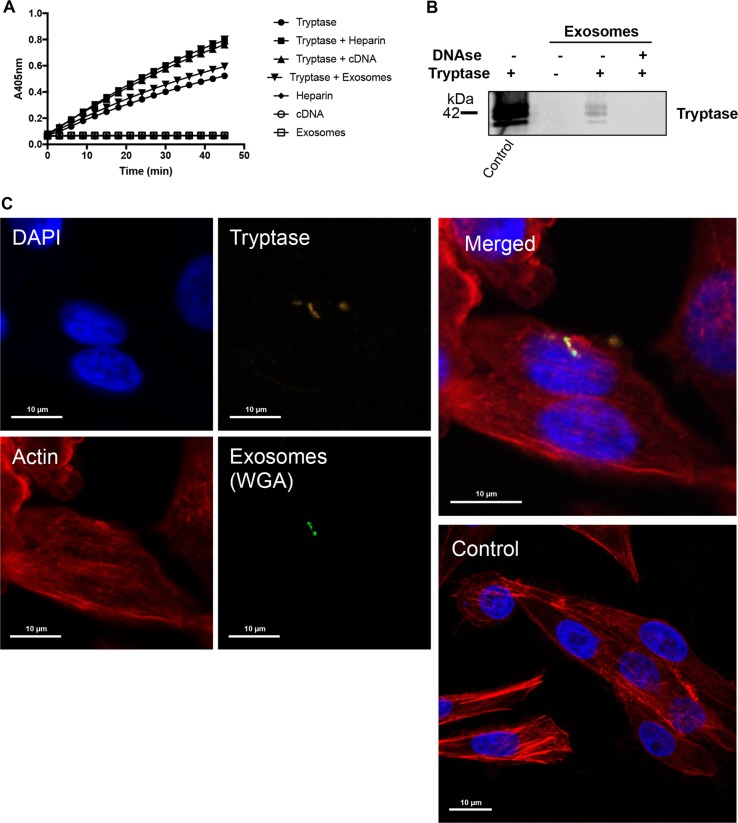
Tryptase is taken up into human melanoma cells as cargo of melanoma cell-derived exosomes. **a** Human tryptase was incubated with heparin, cDNA or purified melanoma-derived exosomes as indicated, followed by assessment of enzymatic activity using a chromogenic substrate (S-2288). Note that the symbols representing samples without tryptase (Heparin, cDNA, exosomes) are superimposed and show no detectable activity against S-2288. **b** Purified melanoma cell-derived exosomes (~4 × 10^7^ exosomes) were either non-treated or digested with DNAse and then incubated with or without tryptase (as indicated), followed by washing and detection of bound tryptase by Western blot analysis. Purified tryptase was included as positive control (left lane). **c** Purified melanoma cell-derived exosomes (~2.5 × 10^7^ exosomes) were labeled with WGA, incubated with purified tryptase, washed and then incubated with human melanoma cells for 6 h, followed by confocal microscopy analysis. Note the presence of tryptase:WGA double-positive exosomes in the nucleus and cytoplasm of the melanoma cells

We next addressed the mechanism of tryptase uptake into the melanoma cells. After staining for tryptase in the melanoma cells we observed that tryptase positivity was largely confined to relatively large entities (Fig. 4c) rather than a diffuse staining, which would be expected if tryptase were freely distributed within the cytosol. This indicates that tryptase is taken up via a mechanism involving larger vehicles and we next asked whether these could represent exosomes. It is known that many types of tumor cells, including melanomas^29^, secrete large amounts of exosomes, and it is thought that exosomes can be exploited by tumor cells as vehicles for cell-cell communication^30^. Tryptase is known to interact with heparin, a highly negatively charged glycosaminoglycan and we have shown that the interaction with heparin is important for promoting the storage of tryptase in MC granules and for enzymatic activity^31–33^. It has been shown that exosomes from certain types of tumor cells can contain DNA^34^ and since DNA, similar to heparin, is a linear polyanionic molecule we hypothesized that tryptase could bind to DNA on exosomes. Further, we considered the possibility that the tryptase uptake into the melanoma cells thereby could be mediated by exosomes. To evaluate this we first assessed whether DNA can potentiate tryptase activity. As seen in Fig. 5a, addition of heparin to tryptase caused an increase in enzymatic activity, and potentiation of enzymatic activity was also seen after addition of DNA. We next assessed whether tryptase can interact with melanoma-derived exosomes (see Supplementary Fig. 4 and Supplementary Table 1 for details of purification) and whether such binding is dependent on exosome-bound DNA. To this end, purified exosomes were incubated with tryptase followed by extensive washing and Western blot analysis to detect exosome-bound tryptase. As seen in Fig. 5b, tryptase was bound to the non-treated exosomes whereas DNA:ase digestion of the exosomes abolished the binding. These findings indicate that tryptase binds to DNA present on the surface of the exosomes and that the enzymatic activity of tryptase is stabilized through this interaction.

To assess whether the uptake of tryptase was mediated by exosomes, we labeled purified exosomes with wheat germ agglutinin (WGA), incubated the WGA-labeled exosomes with tryptase and then added the tryptase-loaded exosomes to melanoma cells. Control experiments showed that WGA-labeled exosomes were vividly taken up by melanoma cells and were found both in the cytoplasm and nucleus (Supplementary Fig. 5). Moreover, tryptase/WGA double-stained exosomes were seen in the interior of the melanoma cells, both in the cytoplasm and in the nucleus, suggesting that tryptase indeed is taken up into the melanoma cells as cargo of exosomes (Fig. 5c). However, it is notable that not all of the melanoma cells were positive for uptake of tryptase-positive exosomes at a given time point.

### Tryptase degrades Lamin B1 and ribonucleoprotein A2/B1 in the nucleus of melanoma cells

**Fig. 6:**
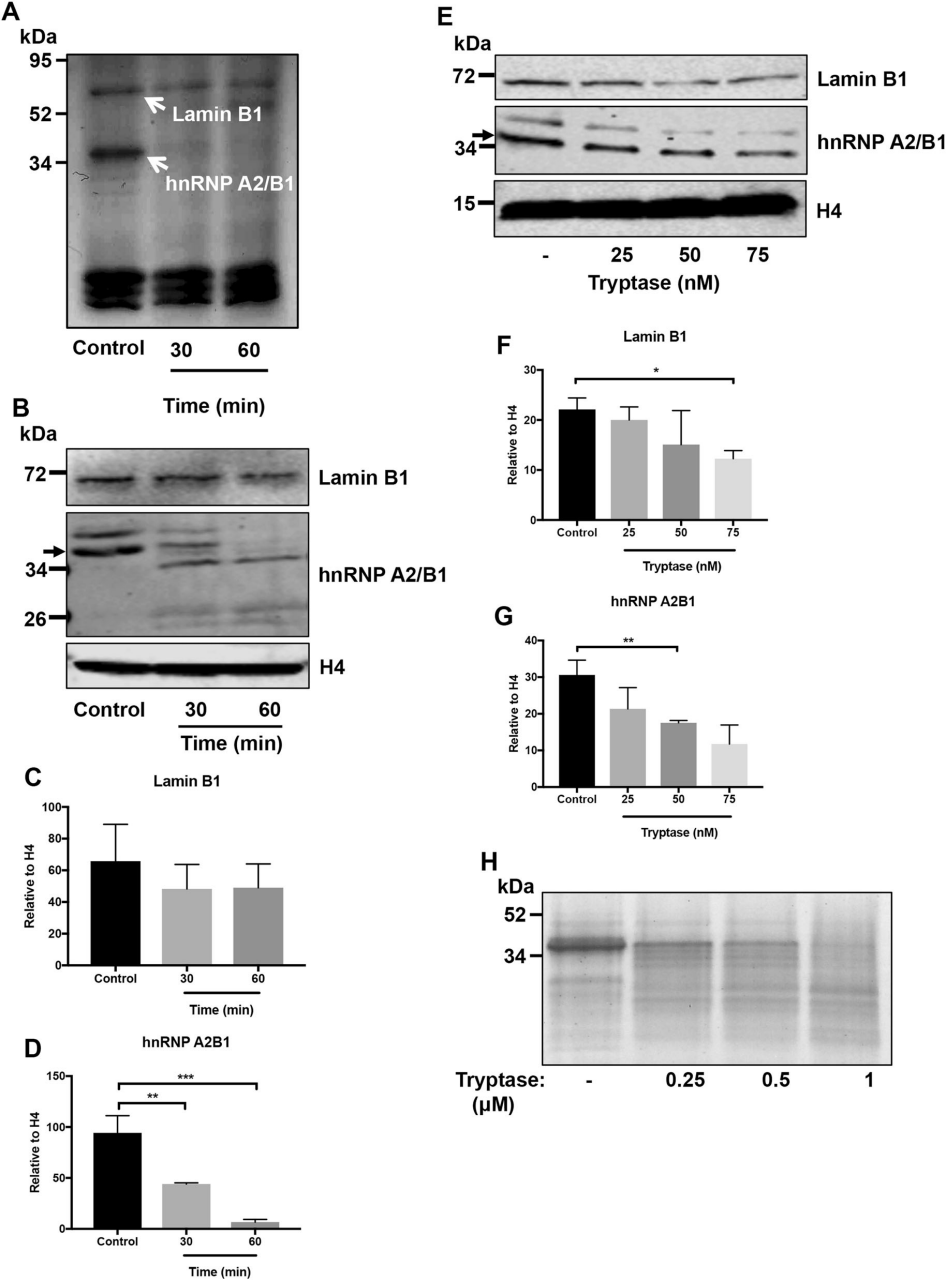
Tryptase degrades nuclear hnRNP A2/B1 and Lamin B1 in human melanoma cells. **a** Nuclear extracts were prepared from human melanoma cells and were incubated with tryptase as indicated, followed by SDS-PAGE analysis and Coomassie staining. The major bands affected by tryptase (arrows) were identified as hnRNP A2/B1 and Lamin B1, respectively. **b**–**d** Western blot analysis of hnRNP A2/B1 and Lamin B1 in tryptase-treated nuclear extracts. Quantification of the Lamin B1 and hnRNP A2/B1 bands by densitometric scanning is shown in (**c**) and (**d**), respectively. **e**–**g** Western blot analysis of hnRNP A2/B1 and Lamin B1 in tryptase-treated cells. Quantification of the Lamin B1 and hnRNP A2/B1 bands by densitometric scanning is shown in (**f**) and (**g**), respectively. **h** Recombinant hnRNP A2/B1 was treated with tryptase, followed by SDS-PAGE and Coomassie staining

Any biological effects of tryptase, being a proteolytic enzyme, are ultimately initiated by proteolysis of one or more target proteins. A key to understanding the mechanism of tryptase action on melanoma cells was therefore to identify the molecular substrate(s) for tryptase. For this purpose we prepared nuclear extracts from the melanoma cells and incubated these with recombinant tryptase, followed by SDS-PAGE analysis. As seen in Fig. 6a, tryptase caused the degradation of two major nuclear proteins of Mw ~70 and ~37 kDa. These compounds were identified by mass spectrometry analysis as Lamin B1 and heterogeneous nuclear ribonucleoprotein A2/B1 (hnRNP A2/B1), respectively. Western blot analysis confirmed that these two proteins were substrates for tryptase (Fig. 6b–g). As a further confirmation of these findings, recombinant tryptase was found to cause extensive degradation of recombinant hnRNP A2/B1 (Fig. 6h).

### Tryptase affects the expression of EGR1 and non-coding RNAs in melanoma cells

**Fig. 7:**
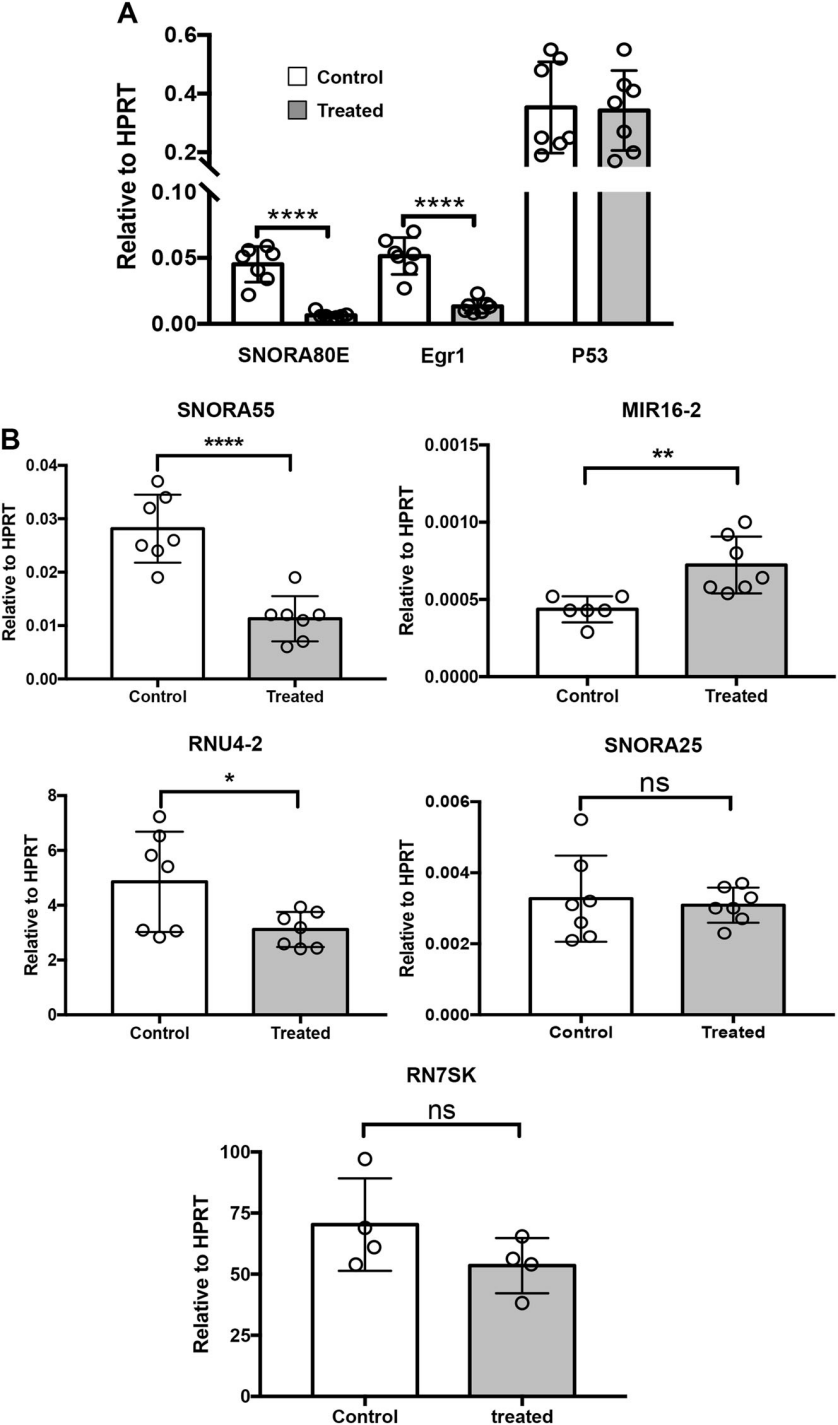
Tryptase affects the expression of non-coding RNAs and EGR1 in human melanoma cells. Human melanoma cells (MEL526) were incubated with 50 nM tryptase for 48 h, followed by qPCR analysis of the expression of SNORA80E, EGR1, P53, SNORA55, MIR16–2, RNU4–2, SNORA25, and RN7SK as indicated. Data are given as mean values ± SEM; *p ≤ 0.05, **p ≤ 0.01, ****p ≤ 0.0001

To further investigate the mechanism by which tryptase impacts on melanoma cells we assessed its impact on gene expression patterns. To this end we used a gene array screening (Supplementary Table 2). This revealed profound effects on numerous genes, a striking finding being that several non-coding RNAs were affected. Among these, SNORA80E, belonging to the small nucleolar RNA family, showed the most profound downregulation. Downregulation was also seen for SNORA55, RNU-2 and SNORA25 whereas MIR16–2 was upregulated. The expression of EGR1 was also downregulated as judged by the gene array screen. To verify these findings, qPCR analysis was conducted. This confirmed a strong and highly significant tryptase-induced downregulation of SNORA83 and EGR1 (Fig. 7a), as well as a significant downregulation of SNORA55 and RNU4–2, and a significant upregulation of MIR16–2 (Fig. 7b). However, the effect on SNORA25 expression could not be confirmed by the qPCR analysis (Fig. 7b).

## Discussion

In malignant settings the immune system is a double-edged sword that can have both anti-tumor effects as well as to assist tumor growth (reviewed in refs. ^35–37^). The anti-tumor mechanisms related to the immune system include the action of cytotoxic T cells, T_H_1 CD4^+^ T cells, NK cells and M1-type macrophages, whereas tumor-promoting effects can be executed by various immune cells such as pro-tumorigenic M2 macrophage subtypes, regulatory T cells, B cells, neutrophils, and T_H_2 CD4^+^ T cells. Here we provide new insight into how tumors can be affected by the immune system, by outlining a novel mechanism by which MCs can suppress melanoma growth.

Our findings reveal a novel principle for regulation of tumor cell proliferation, where a protease secreted from immune cells (MCs) binds to exosomes released from tumor cells, followed by uptake into the nucleus of the target tumor cell. Exosomes are currently being recognized to have strong tumor-promoting features, including promotion of metastasis, invasion, proliferative signaling, activating of angiogenesis and induction of genomic instability^30^. Intriguingly, the findings presented here thus suggest a mechanism where the immune system, represented by MCs, has the ability to hijack the tumor cell-derived exosomes for anti-tumor purposes. Clearly, this opens up novel perspectives as to our understanding of the role of tumor exosomes in malignant settings. As judged by the size and content, the purified vesicles represented exosomes. However, we cannot completely rule out that also other types of extracellular vesicles may have been present to some extent in our exosome preparations.

We show that tryptase enters the nucleus of the target melanoma cells, and that tryptase retains enzymatic activity during this process. Notably, tryptase has a tetrameric organization with the active sites facing a central pore, and due to this organization tryptase is known to resist the action of physiological protease inhibitors^38,39^. Tryptase is thereby well suited for carrying out proteolysis in milieus rich in protease inhibitors. Most likely, this explains how tryptase can maintain enzymatic activity despite having been exposed to protease inhibitors present in the extracellular medium and those present in the cytoplasm and nucleus.

In the nucleus, tryptase was found to degrade Lamin B1. Lamin B1 is a nuclear envelope protein and is known to have major function in maintaining nuclear homeostasis^40^. Degradation of this protein is therefore expected to cause nuclear remodeling and, in agreement with this, we show that melanoma cells exposed to tryptase undergo extensive alterations in the nuclear morphology. Tryptase was also shown to degrade hnRNP A2/B1. hnRNP A2/B1 is a nuclear protein belonging to the hnRNP family, with a known function in regulation of mRNA biogenesis and stability, as well as having an impact on non-coding RNAs^41^. Degradation of hnRNP A2/B1 is thus likely to affect the expression of both mRNAs and non-coding RNAs. Indeed, our findings reveal that treatment of melanoma cells with tryptase has major effects on the expression of numerous genes, in particular the expression of non-coding RNAs. The latter include SNORA80E, a non-coding RNA belonging to the small nucleolar RNA family. Recently it was show that SNORA80E (also denoted SNORA42) is an emerging oncogene in colorectal cancer and in non-small cell lung cancer tumorigenesis^42,43^. Downregulation of SNORA80E expression is therefore expected to dampen tumor cell proliferation. The anti-proliferative activity of tryptase on melanoma cells is thus clearly compatible with suppression of SNORA80E expression. We also noted that tryptase suppressed the expression of SNORA55 and RNU-2. Also these two noncoding RNA species have been implicated as oncogenes^44^ or as prognostic markers of cancer progression^45^, and their suppression can thus contribute to the observed anti-proliferative effects of tryptase on melanoma cells. We also show that tryptase upregulates the expression of MIR16–2. Interestingly, MIR16 is regarded as anti-tumorigenic non-coding RNA species^46^, and its upregulation can thus contribute to the anti-proliferative action of tryptase. In addition to its effects on non-coding RNAs, tryptase was also found to downregulate the expression of EGR1. EGR1 has recently been shown to be oncogenic^47^, and the downregulation of this gene by tryptase could thus add to the anti-melanoma effects of tryptase.

Our findings reveal that tryptase causes clipping of H3, whereas the other nucleosomal histones were not targets for tryptase. H3, like all core histones, has a flexible N-terminal tail protruding out from the nucleosomes, and it is now well established that these N-terminal tails have major functions in regulation of gene expression by being targets for epigenetic modification^48^. Tryptase has previously been shown to cleave off the N-terminal portion of H3^22,23^ and, most likely, the observed tryptase-mediated truncation of H3 in melanoma cells therefore represents cleavage in the N-terminal portion. Removal of the N-terminal portion of H3 will cause a loss of all of the epigenetic marks present in this region, and this is likely to result in effects on gene expression. Possibly, the observed effects of tryptase on gene expression in melanoma cells could thus, at least partly, be attributable to effects of tryptase on H3.

It is intriguing that the combined absence of tryptase, chymase and CPA3 results in protection against melanoma in vivo^16^, whereas we show here that tryptase represents the major direct anti-tumor effector of MCs on melanoma cells. Possibly, chymase and/or CPA3 could have in vivo anti-tumor effects that are not due to direct interaction with melanoma cells, for example by affecting the tumor microenvironment.

Altogether, the findings presented here introduce a novel concept for regulation of tumor cell proliferation. According to our findings, tryptase released from MCs binds to DNA present on the surface of melanoma cell-derived exosomes, followed by transport to the nucleus where tryptase affects multiple nuclear targets, leading to effects on gene expression and decreased proliferation (Supplementary Fig. 6). Further studies will reveal if this mechanism also can be applied to other types of tumor cells and whether the established concept can be utilized for therapeutic purposes. It will also be of interest to assess whether MC mediators other than tryptase can influence melanoma progression. For example, it is known that MCs can express VEGF, thereby potentially promoting tumor angiogenesis^4–8^. MCs are also a rich source of biogenic amines such as histamine and serotonin^12^, which potentially could influence the development of melanomas and other tumors.

## Materials and methods

### Reagents

Rabbit polyclonal anti-histone H2A, H2B, H3 and H4, mouse monoclonal anti-tryptase [AA1], rabbit monoclonal anti-lamin B1, rabbit polyclonal anti-hnRNP A2B1 antibodies were from Abcam (Cambridge, UK). Rabbit polyclonal anti-histone H1 antibody and PrestoBlue^TM^ cell viability reagent were from Invitrogen (Carlsbad, CA). Goat polyclonal to β-actin was from Santa Cruz Biotechnology (Santa Cruz, CA). Rabbit antisera to mMCP6 and CPA3 were as described^49^. Recombinant human skin β-tryptase and human lung β-tryptase were from Promega (Madison, WI) or prepared as described^39^. Recombinant human hnRNP A2B1 was from Abcam. Click-iT^TM^ Plus EdU Pacific Blue^TM^ flow cytometry cell proliferation kit, ActinRed^TM^ 555, ActinGreen^TM^ 488, NucBlue Hoechst 33342 and wheat germ agglutinin Alexa^TM^ 488 were from Molecular Probes (Oregon, OR). AnnexinV-FITC was from BD bioscience (San Jose, CA). DRAQ7^TM^ was from Biostatus (Shepshed, UK). AC 55541 was from Tocris bioscience (Bristol, UK). Dynasore hydrate, Nafamostat mesylate, Heparin sodium salt and Trypan Blue solution were from Sigma-Aldrich (Steinheim, Germany).

### Animals

Wild-type (WT) and tryptase-deficient (mMCP6^−/−^) mice (8–18 weeks old)^20^ were on C57BL/6J genetic background. All animal experiments were approved by the local ethical committee.

### Bone marrow-derived MCs (BMMCs)

Femurs and tibiae from mice of the same gender and age were recovered, and MCs were obtained by culturing bone marrow cells in Dulbecco’s modified Eagle’s medium (DMEM) (SVA, Uppsala, Sweden), supplemented with 30% WEHI-3B conditioned medium, 10% heat-inactivated fetal bovine serum (FBS) (Invitrogen), 50 μg/ml streptomycin sulfate, 60 μg/ml penicillin G, 2 mM L-glutamine (SVA) and 10 ng/ml mouse recombinant IL-3^49^. The cells were kept at 0.5 × 10^6^ cells/ml, at 37 °C in 5% CO_2_; the medium was changed once a week.

### Cell lines

B16F10 mouse melanoma cells were cultured in DMEM supplemented with 10% FBS, 50 μg/ml streptomycin sulfate and 60 μg/ml penicillin G. MEL526 human melanoma cells derived from a non-specified metastatic site were cultured in IMDM supplemented with 2 mM L-glutamine, 1 mM pyruvate, 10% FBS, 50 μg/ml streptomycin sulfate and 60 μg/ml penicillin G. MM466 and MM253 human melanoma cells from metastatic site lymph nodes were cultured in RPMI 1640 supplemented with 2 mM L-glutamine, 25 mM HEPES, 10% FBS, 50 μg/ml streptomycin sulfate and 60 μg/ml penicillin G. All cell lines were kept at 37 °C in 5% CO_2_.

### Coculture of B16F10 and BMMCs

Fractions of 0.5 × 10^6^ B16F10 cells were placed in 15 ml falcon tubes with different concentrations of BMMCs (1:1, 1:2, 1:4 and 1:8) in a total volume of 5 ml in BMMC culture medium. Tubes were submitted to centrifugation (0.4 × *g*, 5 min) and pellets were resuspended in the same medium by pipetting up and down. 6 well culture plates were used for the culture (triplicates). Controls of B16F10 cells alone were prepared using the same culture medium and procedure. Samples were kept at 37 °C in 5% CO_2_ for 48 h. Supernatants were removed and B16F10 confluent cells were washed twice in PBS. The monolayer of B16F10 cells was removed using 0.05% trypsin-EDTA and cells were counted.

### EdU labeling

0.5 ml of 0.1 × 10^6^ cells/ml were distributed individually into a 24-well plate. Cells were treated with 50 nM recombinant human β-tryptase and kept at 37 °C in 5% CO_2_ for 48 h. Inhibitors were added 1 h before addition of tryptase. Two hours before harvesting the cells, 10 μM of EdU was added, supernatants were collected and the monolayer of cells was removed using 0.05% trypsin-EDTA. Cells were then stained using Click-iT^TM^ Plus EdU pacific blue^TM^ flow cytometry kit (Molecular Probes). Flow cytometry analysis was performed (Cytoflex S, Beckman Coulter). Data from 10,000 events/sample were collected and analyzed by the FlowJo software (Ashland, OR).

### Cell fractionation

MEL526 (3 × 10^6^ cells) were collected by centrifugation, washed with PBS and resuspended in 200 μl of 10 mM HEPES, pH 7.9, 10 mM KCl, 1.5 mM MgCl_2_, 0.34 M sucrose and 0.1% Triton X-100. Samples were incubated for 8 min on ice, followed by centrifugation (1300 × *g*, 5 min, 4 °C). The supernatant (cytosolic fraction) was discarded and pellets (nuclear fraction) were washed three times using the same buffer. The pellet was then resuspended in 100 μl of SDS-PAGE sample buffer and analyzed by Western blot.

### Western blot analysis

Samples of 0.5–2 × 10^6^ melanoma cells were collected by centrifugation and immediately solubilized in SDS-PAGE sample buffer. Samples corresponding to equal numbers of cells were subjected to Western blot analysis as previously described^49^. Membranes were scanned using an Odyssey Infrared Imager from Li-Cor (Lincoln, NE).

### Cell viability

MEL526 cells (0.1 × 10^6^ cells/ml) were left untreated or treated with 50 nM recombinant human β-tryptase at 37 °C in 5% CO_2_ for 48 h. Cell viability was measured using the Cell Titer-Blue^®^ cell viability assay (Promega-Invitrogen, Carlsbad, CA) according to the recommendations of the manufacturer or by flow cytometer using AnnexinV-FITC and DRAQ7^TM^.

### Cell size and counts

Aliquots of 10 μl of cell suspension were mixed with the same volume of Trypan Blue solution. Cell size and counts were performed in a cell counter Countess II FL (Life Technologies, Carlsbad, CA).

### Confocal microscopy

Aliquots of 100 μl/well from 50 × 10^5^ cells/ml suspensions were cultured in 8 chamber polystyrene vessel tissue culture treated glass slides (Falcon, New York, NY) overnight or until confluence. The day after cells were left untreated (control) or treated with tryptase. Coculture experiments were performed using the same culture glass slides. Aliquots of 200 μl/well containing B16F10 (50 × 10^5^ cells/ml) together with WT BMMCs (0.4 × 10^6^ cells/ml) (ratio 1:8), were cultured in BMMC medium at 37 °C in 5% CO_2_ for 48 h. At the end of the treatment for both assays, the supernatant was removed carefully and cells were then fixed with 4% paraformaldehyde in PBS for 15 min. One hundred microliters of 50 μg/ml digitonin solution in PBS was added to each individual glass and incubated for 10 min at room temperature. Next, 100 μl anti-tryptase antibody (1:1000) in TBS/1% BSA and/or isotype control at the same concentration were added and left overnight at 4 °C, followed by washing three times with TBS-T. Next, Alexa-conjugated secondary antibody diluted (1:1000) in TBS/1% BSA was added and incubated for 1 h at room temperature. ActinRed/Green^TM^ (Molecular Probes) probes were used to stain cells. Plasma membrane was stained using wheat germ agglutinin Alexa-488 conjugated (Molecular Probes). The slides were kept in the dark, washed three times with TBS-T between each step. Finally, 50 μl of 1 μg/ml DAPI in TBS/1% BSA was added for 2 min, followed by three times washing with TBS-T. The slides were mounted with SlowFade® gold antifade mounting medium (Life Technologies). Samples were analyzed using a laser-scanning microscope equipped with ZEN 2009 software (LSM 710; Carl Zeiss, Berlin, Germany). 3D models were created using Imaris software from Bitplane (Zurich, Switzerland).

### Super-resolution microscopy

Aliquots of 100 μl/well from 50 × 10^5^ cells/ml suspensions were cultured in 8 chamber polystyrene vessel tissue culture treated glass slides (Falcon, New York, NY) overnight or until confluence. Cells were then left untreated (control) or treated with tryptase (50 nM) for 48 h under culture conditions (37 °C in 5% CO_2_). Then, supernatants were removed carefully and cells were fixed with 4% paraformaldehyde in PBS for 15 min. One hundred microliters of 50 μg/ml digitonin solution in PBS was added to each individual glass and incubated for 10 min at room temperature, followed by washing three times with TBS-T. The slides were kept in the dark and 50 μl of 1 μg/ml DAPI in TBS/1% BSA was added for 2 min, followed by three times washing with TBS-T. The slides were mounted with SlowFade® gold antifade mounting medium (Life Technologies). Samples were analyzed using a laser-scanning microscope equipped with ZEN 2009 software (LSM 710 SIM; Carl Zeiss, Berlin, Germany).

### Isolation of exosomes

MEL526 cells were cultured for 3 days at 37 °C in 5% CO_2_. The conditioned medium was harvested and centrifuged at 2000 × *g* for 30 min to remove cells and debris. The supernatant was transferred to a new tube and 0.5 volumes of Total Exosome Isolation Reagent (Invitrogen) was added and mixed by vortexing. Samples were incubated at 4 °C overnight. After incubation, samples were centrifuged at 5000 × *g* for 1 h at 4 °C. The supernatant was discarded and exosomes were washed and resuspended in PBS. Exosome size distribution and concentration was measured using NanoSight LM14C (Malvern Panalytical, Malvern, UK). Aliquots were prepared and stored at −80^o^C until use. The contents of the purified material was analyzed by proteomic analysis, as described^50^.

### Exosome loading and staining

Fifty microliters of exosome suspension in PBS (~4 × 10^7^ exosomes) was incubated with 1 μl of WGA-Alexa 400 (1 mg/ml) for 15 min at room temperature. Samples were washed twice with 1 ml PBS (21,000 × *g*, 45 min, 4 °C) and resuspended in 100 μl PBS. Four microliters of recombinant human skin β-tryptase (200 μg/ml) was added and incubated for 15 min at room temperature. Samples were washed twice with 1 ml PBS (21,000 × *g*, 45 min, 4 °C), and resuspended in 100 μl PBS. Negative controls were prepared in the same way, however, in the absence of β-tryptase and WGA-Alexa 488. Approximately 2.5 × 10^7^ exosomes were added to individual wells (8 well slide) with 200 μl of 0.5 × 10^5^ MEL526 cells for 6 h, followed by confocal microscopy analysis.

### 3D models

Confocal Z-stack pictures were used to create 3D images and videos with Imaris – Microscopy Image Analysis Software (Bitplane AG, Zurich, Switzerland).

### Quantitative real time RT-PCR

Total RNA preparation and quantitative real-time PCR (qPCR) were performed as previously described^49^. The following primers were used:

#### Murine

Hprt Forward 5′-GAT TAG CGA TGA TGA ACC AGG TTA-3′; Hprt Reverse 5′-GAC ATC TCG AGC AAG TCT TTC AGT C-3′; Dct2 Forward 5′-TCC TCC ACT CTT TTA CAG ACG-3′; Dct2 Reverse 5′-ATT CGG TTG TGA CCA ATG GG-3′; GP100 Forward 5′-AGC ACC TGG AAC CAC ATC TA-3′; GP100 Reverse 5′-CCA GAG GGC GTT TGT GTA GT-3′.

#### Human

HPRT Forward 5′-TGG AGT CCT ATT GAC ATC GCC-3′; HPRT Reverse 5′-AAC AAC AAT CCG CCC AAA GGG-3′; SNORA80E Forward 5′- TGG ATT TAT GGT GGG TCC TTC TCT G-3′; SNORA80E Reverse 5′- CAG GTA AGG GGA CTG GGC AAT GGT T-3′; EGR1 Forward 5′- ACC CCT CTG TCT ACT ATT AAG GC-3′; EGR1 Reverse 5′-TGG GAC TGG TAG CTG GTA TTG-3′; P53 Forward 5′-GTG CGT GTT TGT GCC TGT CC-3′; P53 Reverse 5′-GTG CTC GCT TAG TGC TCC CT-3′; SNORA55 Forward 5′-ACC TGA ATC TTT CCC ATT CCT T-3′; SNORA55 Reverse 5′-CTG GAT TTC CTC TGC TCA TTC T-3′; MIR16–2 Forward 5′-CCA CTC TAG CAG CAC GTA ATT-3′; MIR16–2 Reverse 5′-TCA CAC TAA AGC AGC ACA GTA A-3′; RNU4–2 Forward 5’-TCG TAG CCA ATG AGG TTT ATC C-3′; RNU4–2 Reverse 5′-GCC AAT GCC GAC TAT ATT TCA AG-3′; SNORA25 Forward 5′-GGG CTT ATG AGG CTG TGA AA-3′; SNORA25 Reverse 5′-AGG AGT GCT ATG GCT TCC TA-3′.

### Statistical analysis

Data were analyzed by either Student’s *t*-test or by Two-way ANOVA using GraphPad Prism 7 software (La Jolla, CA) and are presented as mean values ± SEM; *p < 0.05, **p < 0.01, ***p < 0.001, ****p < 0.0001.

## Supplementary information

Suppl Table 1

Suppl Table 2

Suppl Fig 1

Suppl Fig 2

Suppl Fig 3

Suppl Fig 4

Suppl Fig 5

Suppl Fig 6

Suppl Video 1

Suppl Video 2
