# Supplementary material for: Exosome-mediated uptake of mast cell tryptase into the nucleus of melanoma cells: a novel axis for regulating tumor cell proliferation and gene expression
Source: Cell Death Dis. 2019 Sep 10;10(9):659. doi: 10.1038/s41419-019-1879-4 (PMC6736983; doi:10.1038/s41419-019-1879-4)
Supplement: Supplementary file 1 — Suppl Table 1 [file 41419_2019_1879_MOESM1_ESM.docx]

**Suppl. Table 1.** Proteomic analysis of melanoma cell-derived exosomes. The table lists proteins that are markers of exosomes, as specified in the ExoCarta exosome database (www.exocarta.org/). Cytochrome C (mitochondrial marker) and Lamin B1 (nuclear marker) were not detected.

| **Accession** | **Gene symbol** | **Score** | **ExoCarta (ranking)** |
| --- | --- | --- | --- |
| P60709 | ACTB | 382,5 | 4 |
| P04406 | GAPDH | 232,7 | 3 |
| P06733 | ENO1 | 196,7 | 8 |
| P11142 | HSPA8 | 193,3 | 1 |
| P07900 | HSP90AA1 | 170 | 9 |
| P68104 | EEF1A1 | 132,62 | 10 |
| P07355 | ANXA2 | 142 | 7 |
| E9PJK1 | CD81 | 63,8 | 6 |
| A6NNI4 | CD9 | 22,8 | 2 |
| F8VNT9 | CD63 | 2,81 | 5 |
