# Supplementary material for: Exosome-mediated uptake of mast cell tryptase into the nucleus of melanoma cells: a novel axis for regulating tumor cell proliferation and gene expression
Source: Cell Death Dis. 2019 Sep 10;10(9):659. doi: 10.1038/s41419-019-1879-4 (PMC6736983; doi:10.1038/s41419-019-1879-4)
Supplement: Supplementary file 2 — Suppl Table 2 [file 41419_2019_1879_MOESM2_ESM.docx]

**Suppl. Table 2.** Effects of tryptase on gene expression in human MEL526 melanoma cells. Affymetrix gene chip analysis, showing genes that were affected by tryptase.

| Gene Symbol | Gene description | Fold-change induced by tryptase  (Log 2 fold change) | Expression (arb units)  control | Expression (arb units)    tryptase |
| --- | --- | --- | --- | --- |
| SNORA80E | small nucleolar RNA, H/ACA box 80E | -3,759598 | 10,47533 | 6,715732 |
| EGR1 | early growth response 1 | -2,256714 | 8,023516 | 5,766802 |
| MIR548AI | microRNA 548ai | -2,105401 | 10,89253 | 8,787129 |
| RNU6-844P | RNA, U6 small nuclear 844 | -2,02611 | 5,84474 | 3,81863 |
| MIR16-2 | microRNA 16-2 | 1,871781 | 5,096029 | 6,96781 |
| RNU6-297P | RNA, U6 small nuclear 297 | -1,851165 | 9,432159 | 7,580994 |
| OR5AW1P | olfactory receptor family 5 subfamily AW member 1 | -1,828212 | 5,00757 | 3,179358 |
| SNORD111 | Small nucleolar RNA SNORD111 [ | -1,80316 | 5,904946 | 4,101786 |
| MIR626 | microRNA 626 | -1,773505 | 4,719987 | 2,946482 |
| SNORA80E | microRNA 548bb | -1,76719 | 5,011616 | 3,244426 |
| EGR2 | --- | 1,750164 | 2,864728 | 4,614892 |
| MIR548AI | --- | -1,742193 | 5,091682 | 3,349489 |
| RNU6-844P | microRNA 548t | -1,732702 | 7,134684 | 5,401982 |
| MIR16-3 | Small nucleolar RNA SNORA25 | 1,716562 | 3,411299 | 5,127861 |
| AC099506.2 | small nucleolar RNA, H/ACA box 55 | 1,704773 | 3,864526 | 5,569299 |
| RNU6-297P | RNA, U4 small nuclear 2 | -1,699733 | 5,978806 | 4,279073 |
| AC069286.2 | killer cell lectin-like receptor subfamily F, member 2 | -1,69327 | 8,444282 | 6,751012 |
| AL080285.2 | RNA, U6 small nuclear 697, | 1,681909 | 3,179218 | 4,861127 |
| RNA5SP88 | apolipoprotein O pseudogene 1 [ | -1,67366 | 12,70441 | 11,03075 |
| RP11-1058G23.2 | RNA, U4 small nuclear 87, | -1,66262 | 12,45411 | 10,79149 |
| SNORD112 | --- | 1,66167 | 2,919093 | 4,580763 |
| MIR627 | envoplakin-like | -1,65493 | 5,804002 | 4,149072 |
| SNORA80E | small nucleolar RNA, H/ACA box 80E | -1,612241 | 5,463097 | 3,850856 |
