# Supplementary material for: Exosome-mediated uptake of mast cell tryptase into the nucleus of melanoma cells: a novel axis for regulating tumor cell proliferation and gene expression
Source: Cell Death Dis. 2019 Sep 10;10(9):659. doi: 10.1038/s41419-019-1879-4 (PMC6736983; doi:10.1038/s41419-019-1879-4)
Supplement: Supplementary file 3 — Suppl Fig 1 [file 41419_2019_1879_MOESM3_ESM.pdf]

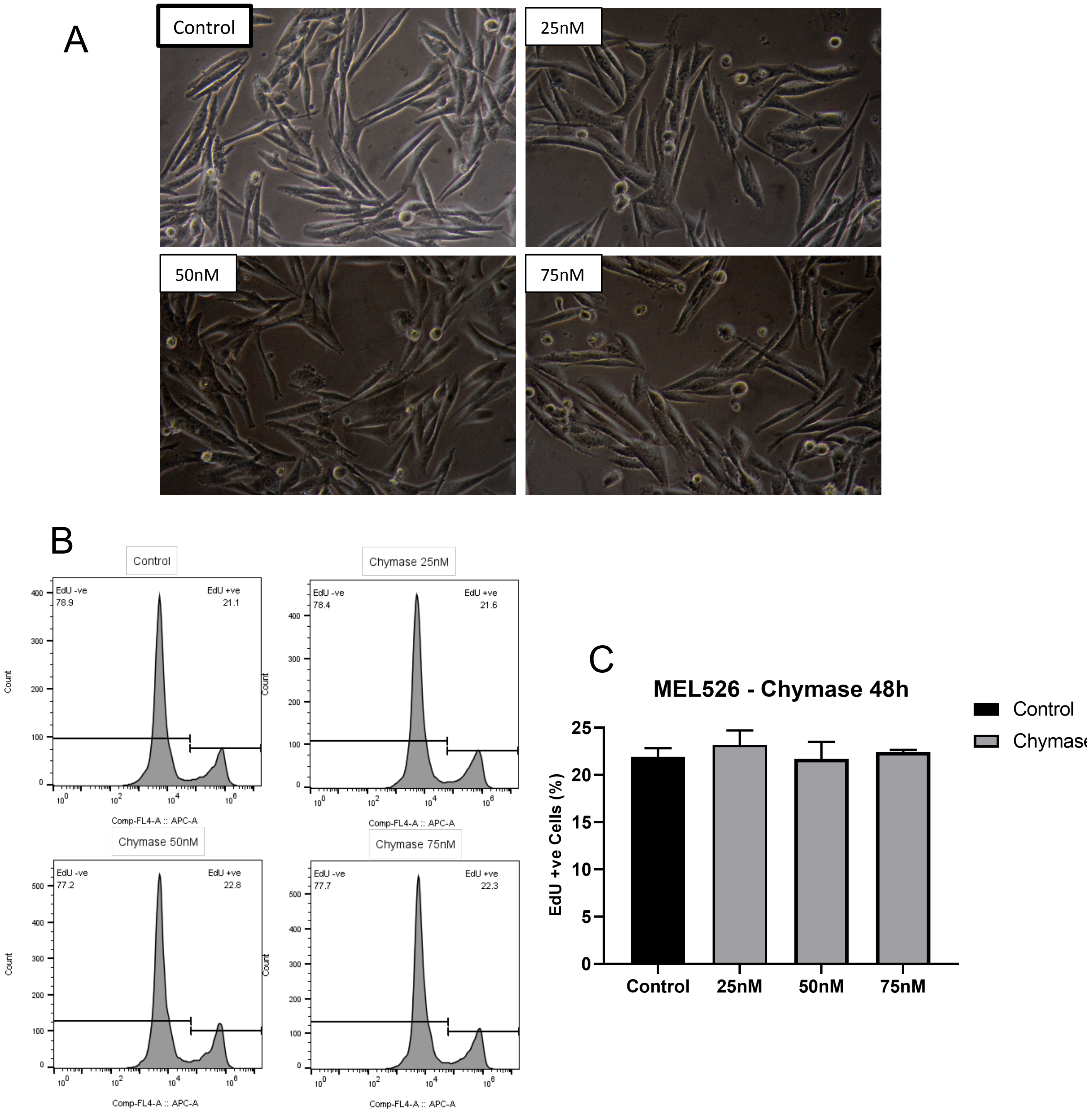

Suppl. Fig 1. Chymase does not affect the morphology or proliferation of melanoma cells. Human melanoma cells (MEL526) were incubated with 25-75 nM chymase for 48 hours. (A) Microscopy analysis of control and chymase-treated MEL526 cells. (B) Chymase-treated melanoma cells (MEL526) were assessed for proliferation through EdU staining. (C) Quantification of EdU staining of MEL526. Data are given as mean values ± SEM (n = 3). Note that chymase does not affect the morphology or proliferation of MEL526 cells.
