# Supplementary material for: Exosome-mediated uptake of mast cell tryptase into the nucleus of melanoma cells: a novel axis for regulating tumor cell proliferation and gene expression
Source: Cell Death Dis. 2019 Sep 10;10(9):659. doi: 10.1038/s41419-019-1879-4 (PMC6736983; doi:10.1038/s41419-019-1879-4)
Supplement: Supplementary file 4 — Suppl Fig 2 [file 41419_2019_1879_MOESM4_ESM.pdf]

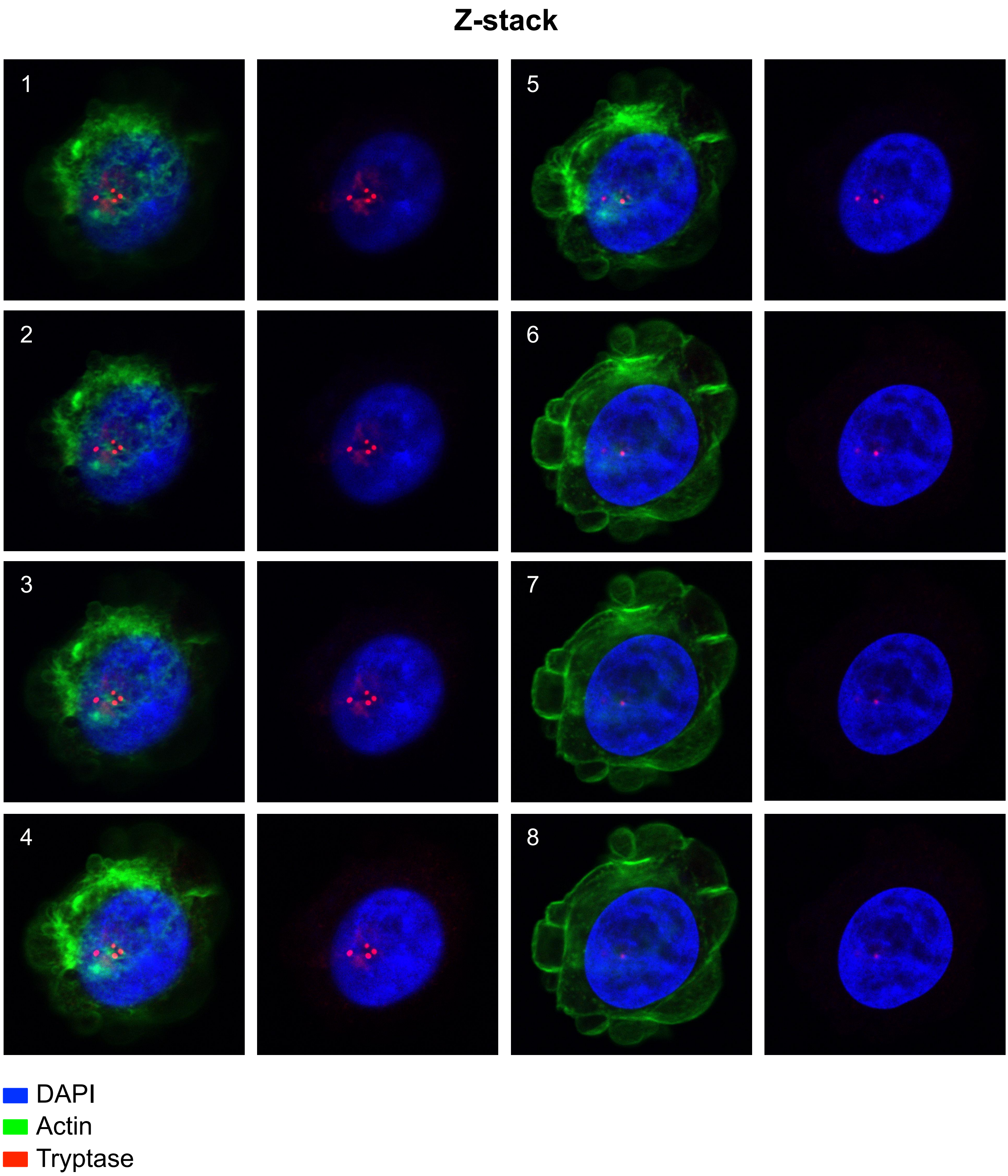

Suppl. Fig 2. Nuclear localization of human tryptase in melanoma cells. Human melanoma cells (MEL526) were incubated with 50 nM tryptase overnight, followed by confocal microscopy analysis. The figure shows a Z-stack analysis revealing a nuclear localization of tryptase.
