# Supplementary material for: Exosome-mediated uptake of mast cell tryptase into the nucleus of melanoma cells: a novel axis for regulating tumor cell proliferation and gene expression
Source: Cell Death Dis. 2019 Sep 10;10(9):659. doi: 10.1038/s41419-019-1879-4 (PMC6736983; doi:10.1038/s41419-019-1879-4)
Supplement: Supplementary file 5 — Suppl Fig 3 [file 41419_2019_1879_MOESM5_ESM.pdf]

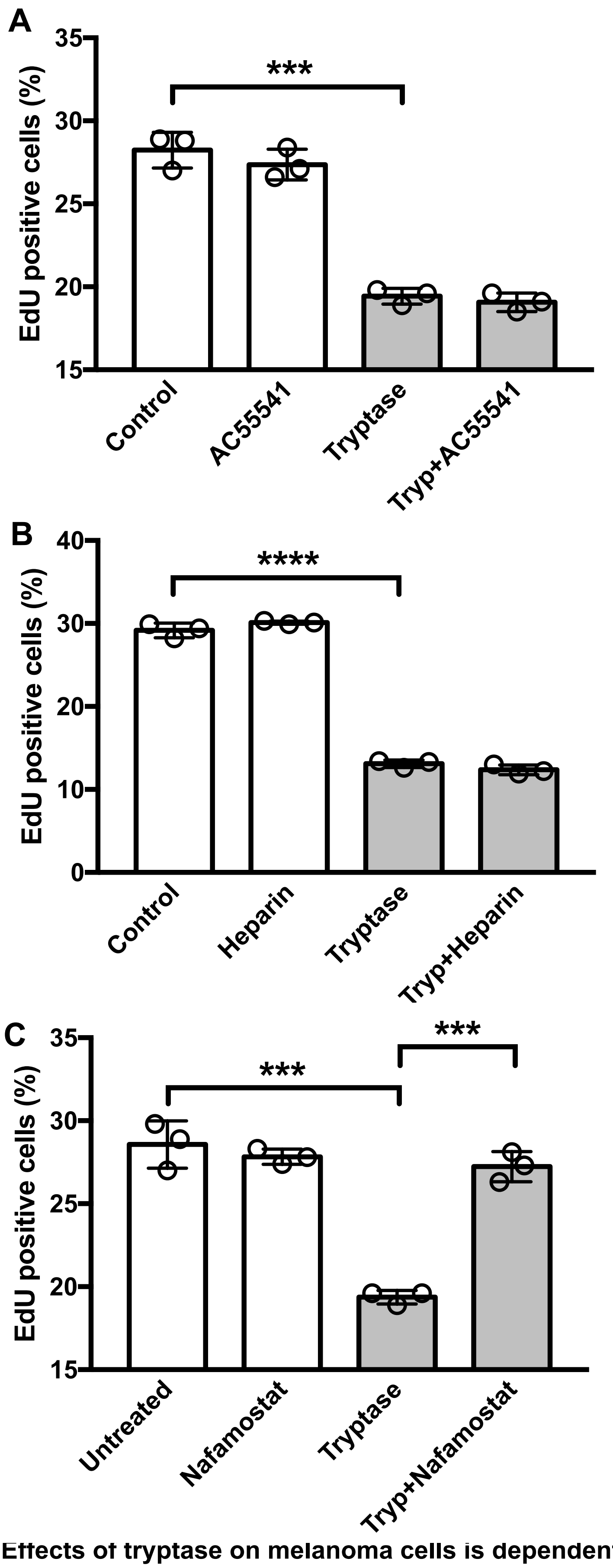

Suppl. Fig. 3. Effects of tryptase on melanoma cells is dependent on intact enzymatic activity but is independent of PAR-2 and heparin. Human melanoma cells (MEL526) were incubated with 50 nM tryptase for 48 h, followed by EdU staining. The effects of (A) a PAR-2 agonist (AC5554; 5 µM), (B) heparin (100 µg/ml) and (C) a selective tryptase inhibitor (Nafamostat; 5 µM) on EdU positivity was assessed. Results are given as mean values ± SEM; *** $p \leq 0.001$, ****, $p \leq 0.0001$.
