# Supplementary material for: Exosome-mediated uptake of mast cell tryptase into the nucleus of melanoma cells: a novel axis for regulating tumor cell proliferation and gene expression
Source: Cell Death Dis. 2019 Sep 10;10(9):659. doi: 10.1038/s41419-019-1879-4 (PMC6736983; doi:10.1038/s41419-019-1879-4)
Supplement: Supplementary file 6 — Suppl Fig 4 [file 41419_2019_1879_MOESM6_ESM.pdf]

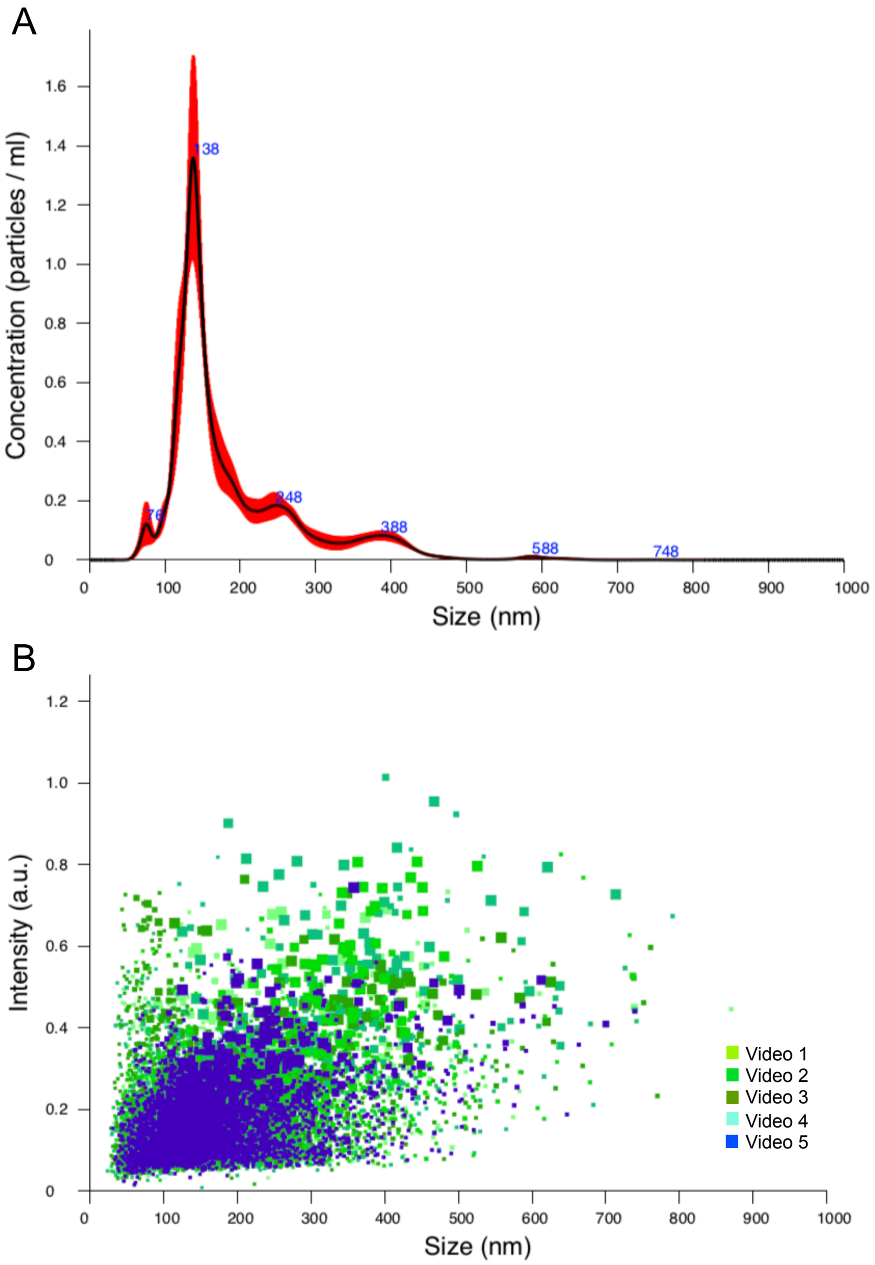

Suppl. Fig 4. Purification of exosomes from melanoma cells. Human melanoma cells (MEL526) were cultured for 72 h. The conditioned medium was recovered and used for exosome isolation using the total exosome isolation reagent from cell culture media. Exosome analysis was performed using NanoSight LM14C. Figures are presenting data from 5 videos of 60 seconds. (A) Particles size distribution. (B) Size/intensity analyses.
