# Supplementary material for: Exosome-mediated uptake of mast cell tryptase into the nucleus of melanoma cells: a novel axis for regulating tumor cell proliferation and gene expression
Source: Cell Death Dis. 2019 Sep 10;10(9):659. doi: 10.1038/s41419-019-1879-4 (PMC6736983; doi:10.1038/s41419-019-1879-4)
Supplement: Supplementary file 7 — Suppl Fig 5 [file 41419_2019_1879_MOESM7_ESM.pdf]

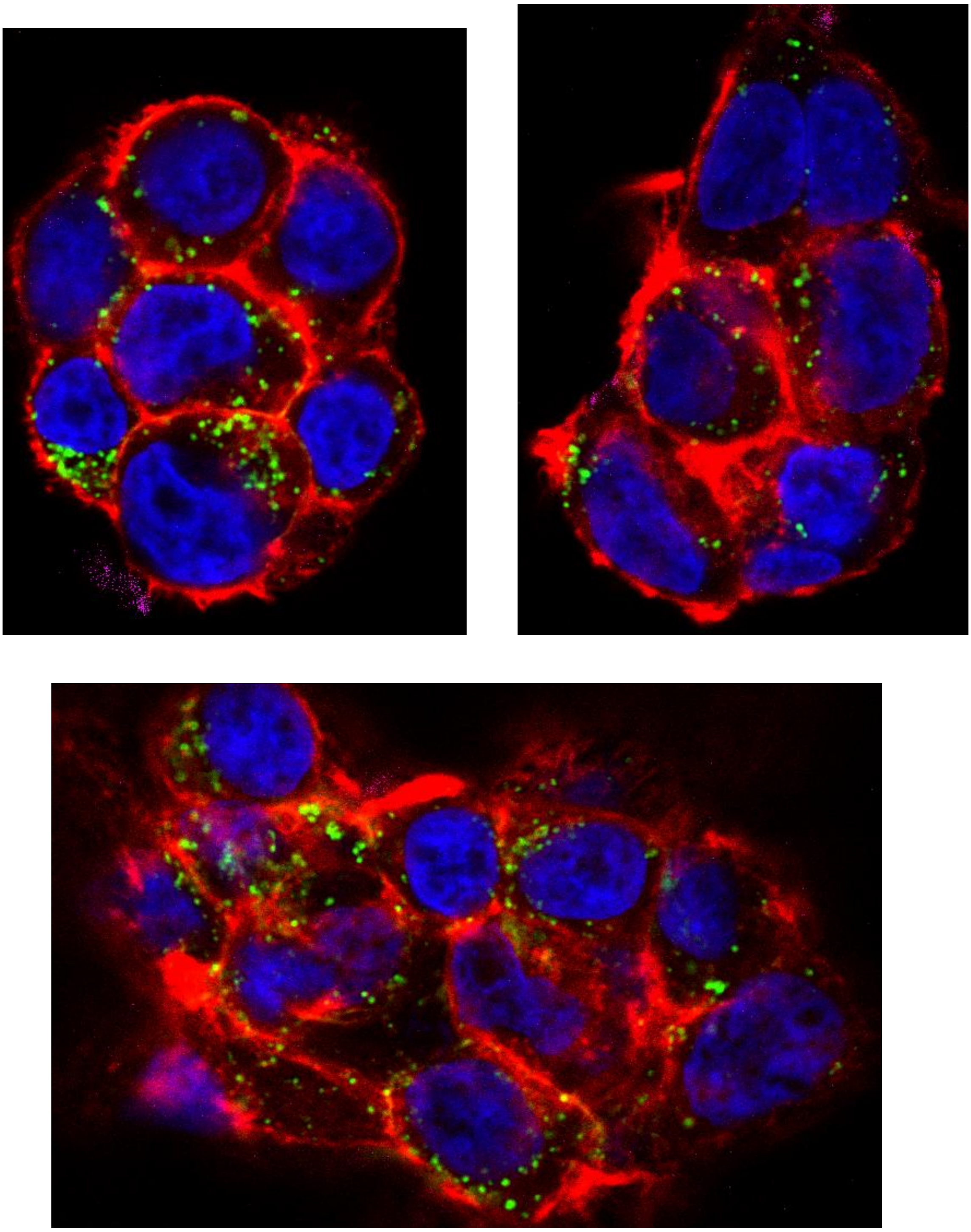

Suppl. Fig 5. **Uptake of exosomes by melanoma cells**. Exosomes were purified from human melanoma cells (MEL526) using the total exosome isolation reagent from cell culture media and were labeled with Wheat Germ Agglutinin (WGA) Alexa^{TM} 488. WGA –labeled exosomes were incubated with melanoma cells overnight. Cells were washed and assessed for uptake of WGA-labeled exosomes.
