# Supplementary material for: Exosome-mediated uptake of mast cell tryptase into the nucleus of melanoma cells: a novel axis for regulating tumor cell proliferation and gene expression
Source: Cell Death Dis. 2019 Sep 10;10(9):659. doi: 10.1038/s41419-019-1879-4 (PMC6736983; doi:10.1038/s41419-019-1879-4)

Suppl. Fig. 6. Model for how mast cell tryptase affects melanoma cells. Melanoma cells secrete exosomes coated with DNA. Mast cells located in the vicinity of melanoma cells secrete tryptase bound to proteoglycans (serglycin) from secretory granules. Tryptase binds to melanoma-derived exosomes by interacting with DNA, followed by uptake into the melanoma cells by endocytosis. Tryptase enters the nucleus and causes proteolysis of several nuclear targets, leading to nuclear remodeling, effects on gene expression and reduced proliferation

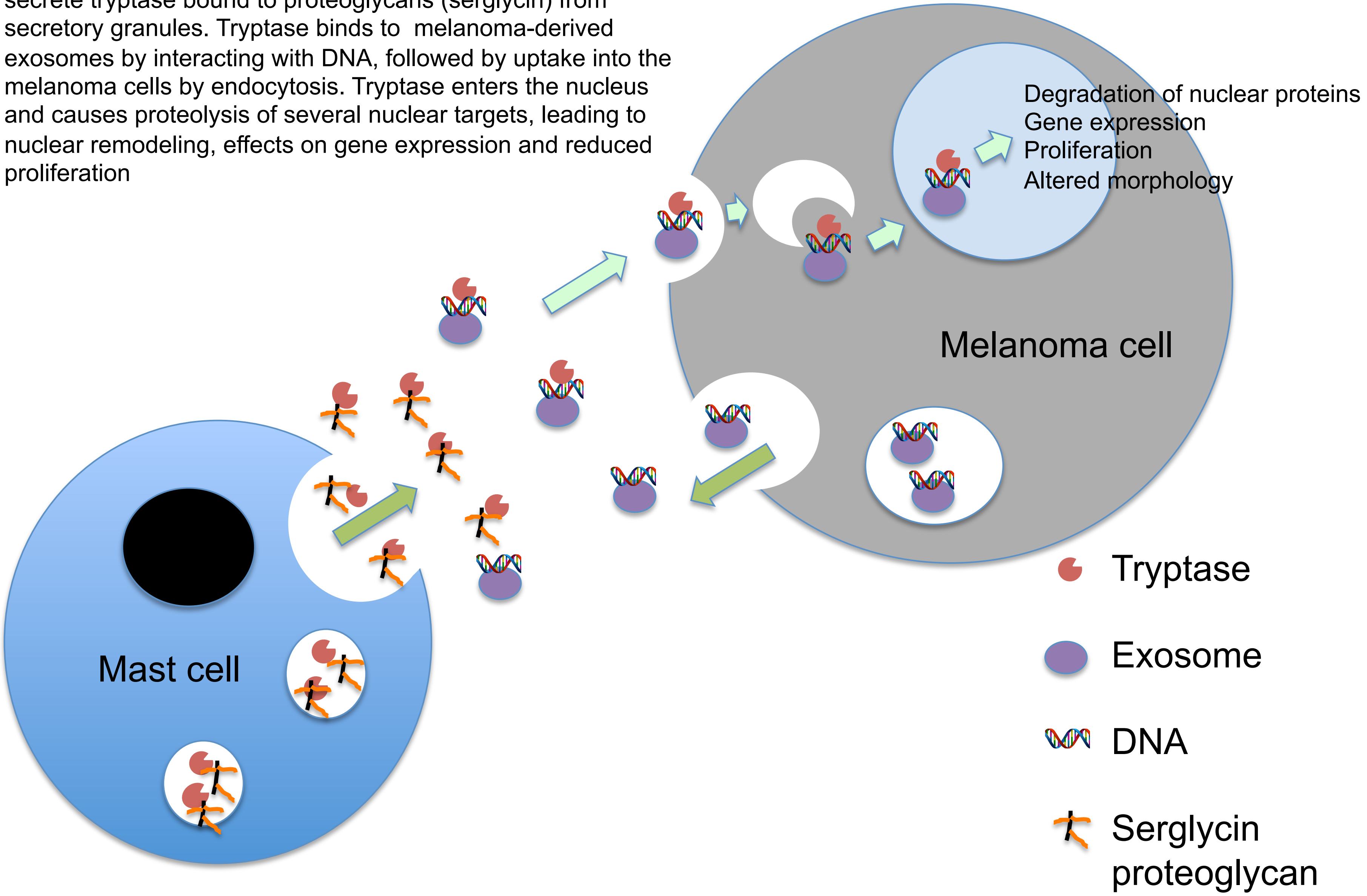

Supplement: Supplementary file 8 — Suppl Fig 6 [file 41419_2019_1879_MOESM8_ESM.pdf]
